# Conversational AI and Personal Growth: Insights from a Critical Integrative Review

**DOI:** 10.3390/bs16050756

**Published:** 2026-05-12

**Authors:** Shivali Sharma, Pranika Vohra, Laura M. Vowels

**Affiliations:** 1 School of Psychology, University of Roehampton, London SW15 4JD, UK; shivali.sharma@roehampton.ac.uk; 2 Department of Psychology, North Dakota State University, Fargo, ND 58108, USA; pranika.vohra@ndsu.edu

**Keywords:** conversational AI, chatbots, self-development, personal growth, therapeutic alliance, attachment

## Abstract

Conversational AI systems are increasingly integrated into individuals’ emotional and relational lives, yet whether such interactions can meaningfully support personal growth remains poorly understood. This critical integrative review synthesises theoretical frameworks from humanistic psychology, self-determination theory, attachment theory, and relationship science with empirical research on human-AI interaction to address this question directly. Drawing on 130 studies spanning therapeutic, companion, and educational AI contexts, the review identifies four interdependent domains that together shape growth outcomes in human-AI contexts: user-related characteristics, AI design features, human-AI relational dynamics, and broader contextual factors. The evidence supports a position of bounded optimism: conversational AI can scaffold early emotional stabilisation, structured self-reflection, and therapeutic skill rehearsal, yet it remains structurally limited in replicating the reciprocal vulnerability, rupture-and-repair processes, and calibrated ideal-self affirmation that underpin enduring psychological development. Engagement-optimised design—including flattery, progressive intimacy escalation, and unconditional validation—is consistently identified as a systematic barrier to growth across multiple domains of the framework. An integrative four-domain conceptual framework is proposed to guide both future research and the ethical design of AI systems that support, rather than undermine, the relational mechanisms fundamental to human flourishing.

## 1. Introduction

Decades of psychological research have established that personal growth—the sustained process by which individuals develop greater self-awareness, emotional integration, autonomy, and alignment with personally valued goals—is fundamentally relational in nature: it emerges through close relationships that provide emotional safety, honest feedback, and opportunities for genuine self-expansion (Feeney & Collins, 2015; Koole et al., 2019; Maurer & Daukantaitė, 2020). Conversational AI systems—computer-based systems that engage users in natural language dialogue to simulate interactive, goal-directed, or socially responsive communication—are now woven into the everyday emotional and relational lives of millions of people worldwide, yet whether interactions with these systems can meaningfully support personal growth remains poorly understood. Conversational AI systems offer a relational context that is structurally unlike anything humans have previously encountered at scale: always available, never tired, non-judgmental, and increasingly capable of extended emotional dialogue (Brandtzaeg et al., 2022; Malfacini, 2025). Understanding whether and how this new relational environment participates in, or undermines, the processes through which people grow is therefore an important theoretical and practical question for psychology. The present review addresses the following question: under what conditions, if any, can interactions with conversational AI meaningfully support personal growth? A related question concerns which theoretical traditions on intra- and interpersonal processes of personal growth speak most directly to human–AI relationships. Below, we review the specific theoretical frameworks relevant to personal growth and examine how each may or may not apply to human–AI contexts.

## 2. Theoretical Foundations: Personal Growth and Human–AI Interaction

Humanistic psychology provides an early theoretical account of how humans achieve personal growth. Rogers’ organismic valuing perspective proposes that individuals possess an inherent actualising tendency directed toward authenticity, integration, and fulfilment of potential (Maurer & Daukantaitė, 2020). This developmental tendency unfolds most effectively in environments characterised by empathy, congruence, and unconditional positive regard, which together create the psychological safety required for open self-exploration and emotional integration (Maurer & Daukantaitė, 2020). Under such conditions, individuals become increasingly capable of incorporating new experiences without defensive distortion, thereby enabling movement toward more coherent and authentic self-structures (Maurer & Daukantaitė, 2020). Conversational AI systems can, in principle, approximate some of these conditions—offering consistent positive regard, non-judgmental engagement, and a low-risk space for self-expression.

Self-Determination Theory (SDT) proposes that wellbeing and healthy development depend on meeting three basic psychological needs—autonomy, competence, and relatedness—and that social environments which support these needs encourage motivated, self-directed growth (Ryan & Deci, 2000; Koole et al., 2019). AI systems have some capacity to meet these needs: they can support skill-building (competence), allow users to direct their own conversations (autonomy), and provide a sense of being heard and connected (relatedness) (Deng & Yu, 2023; Fitzpatrick et al., 2017; Brandtzaeg et al., 2022). Whether these effects are lasting, and whether they complement or crowd out need satisfaction in real human relationships, are questions this review addresses.

Beyond intrapsychic processes, relationship science demonstrates that personal development is fundamentally embedded within interpersonal systems (Feeney & Collins, 2015; Purol & Chopik, 2024). Attachment theory further specifies that close relationships support growth through secure-base and safe-haven functions, enabling exploration, goal pursuit, and emotional regulation across the lifespan (Feeney & Collins, 2015; Fraley, 2019). In human relationships, secure-base support facilitates engagement with challenges by providing a reliable backdrop of encouragement and availability, while safe-haven support provides comfort and reassurance during periods of distress. Emerging evidence suggests that conversational AI can approximate aspects of both functions: users report emotional comfort and perceived non-judgmental support, and AI systems may also scaffold exploration by prompting reflection, encouraging goal-directed thinking, and providing structured guidance for problem-solving (Kasturiratna & Hartanto, 2025; Xie & Pentina, 2022). Recent work has begun to examine these dynamics more explicitly within human–AI relationships, including the development of measures capturing attachment-related processes in AI interactions (Kasturiratna & Hartanto, 2025).

The self-expansion model proposes that individuals grow by incorporating the perspectives, resources, and identities of close others into the self-concept through shared novel experiences (Aron et al., 2022). Complementary work on the Michelangelo phenomenon shows that growth toward one’s ideal self depends on calibrated affirmation from partners who respond to aspirational rather than actual self-presentations (Rusbult et al., 2009). These models are harder to apply to Conversational AI: self-expansion presupposes a genuinely autonomous other whose perspectives differ from one’s own (Lamarche, 2022), and the Michelangelo phenomenon requires a partner capable of accurate calibration rather than unconditional validation—a particular challenge for Conversational AI systems that are commercially incentivised toward user satisfaction and sycophantic agreement (Fang et al., 2025; Shevlin, 2024).

Across these theoretical traditions, personal growth is relationally scaffolded, emerging through environments that provide emotional safety, affirmation, autonomy support, and opportunities for exploration (Aron et al., 2022; Feeney & Collins, 2015; Koole et al., 2019; Maurer & Daukantaitė, 2020; Rusbult et al., 2009). They provide a foundation for examining how emerging AI contexts may participate in these same processes. For the purposes of this review, personal growth is defined as a sustained developmental process characterised by increasing self-awareness, greater integration of experience into a coherent and authentic self-structure, expanding autonomy and volitional self-direction, and progressive movement toward personally valued goals and identity commitments (Koole et al., 2019; Maurer & Daukantaitė, 2020). Outcomes frequently reported in the conversational AI literature—such as symptom reduction, self-disclosure, engagement, and help-seeking—are not treated as indicators of personal growth in themselves, but as proximal processes that may co-occur with, or contribute to, developmental change. Accordingly, this review draws on studies examining these outcomes where they provide insight into mechanisms relevant to growth, while maintaining a distinction between short-term psychological improvement and broader developmental progress. In this sense, improvements in symptoms or engagement are considered neither sufficient nor irrelevant to personal growth, but as potential pathways through which growth-related processes may be initiated or supported.

## 3. Conversational AI in Emotional and Relational Contexts

Conversational AI systems span a wide range of technologies—from rule-based therapeutic chatbots delivering structured CBT protocols to generative LLM-based companions and domain-specific educational agents—differing substantially in architecture, relational affordances, and the psychological processes they activate (Fitzpatrick et al., 2017; Siddals et al., 2024; Malfacini, 2025). Where evidence derives specifically from one system type, this is noted in the synthesis; the review’s primary aim is to identify mechanisms that cut across conversational AI types. Such systems are used for reflective dialogue, mood regulation, problem-solving, and companionship in both clinical and non-clinical settings (Brandtzaeg et al., 2022; Fitzpatrick et al., 2017; Zhou et al., 2020). Their accessibility, anonymity, and continuous availability position conversational AI as a scalable alternative or supplement to traditional mental health services, particularly for individuals who experience barriers to accessing care (Kretzschmar et al., 2019; Laranjo et al., 2018).

Empirical research suggests that users frequently perceive conversational agents as supportive and emotionally responsive (Sorin et al., 2023). Studies of therapeutic chatbots such as Woebot indicate reductions in anxiety and depressive symptoms alongside high levels of user engagement (Fitzpatrick et al., 2017). Research specifically examining chatbots in the context of relationship support has found that users rate AI responses as more empathic and helpful than those of human relationship experts, and that qualified relationship therapists rate single chatbot-delivered sessions highly on empathy, active listening, and exploration (Vowels, 2024; Vowels et al., 2025a). Users interacting with conversational AI more broadly often report feelings of empathy, validation, and psychological comfort during interactions, suggesting that conversational AI systems may evoke relational experiences that resemble aspects of therapeutic alliance (Siddals et al., 2024).

Self-disclosure represents a key mechanism underlying these interactions (A. Ho et al., 2018; Kretzschmar et al., 2019). The perceived non-judgmental nature of AI conversations can reduce social evaluation concerns, enabling users to disclose personal thoughts and emotions more readily than they might in human contexts (A. Ho et al., 2018; Kretzschmar et al., 2019). Increased disclosure may support early emotional processing and self-reflection for individuals who might otherwise avoid personal disclosure due to stigma or social anxiety (Kretzschmar et al., 2019). Emerging research further suggests that interactions with conversational AI may influence how individuals perceive relationships and emotional support. Studies examining human–AI relationships indicate that users can develop perceptions of companionship, empathy, and relational responsiveness toward conversational agents (Brandtzaeg et al., 2022; Skjuve et al., 2022).

At the same time, conversational AI differs fundamentally from human relationships in several important respects. Unlike human partners or therapists, AI systems lack genuine agency, emotional experience, and reciprocal vulnerability. As a result, AI interactions may struggle to replicate key relational mechanisms—such as conflict, rupture-and-repair cycles, and mutual emotional influence—that contribute to long-term developmental change in human relationships (Lamarche, 2022; Feeney & Collins, 2015).

Research examining patterns of engagement further suggests that outcomes may vary depending on how conversational AI systems are used. For some individuals, interactions with AI function as a form of reflective dialogue that complements existing relationships and supports emotional regulation (Brandtzaeg et al., 2022). For others, particularly those experiencing loneliness or social isolation, conversational AI may serve as a form of companionship that partially substitutes for human interaction (Zhou et al., 2020; Malfacini, 2025). Although such interactions may provide immediate emotional comfort, sustained reliance on AI companionship may also reshape relational expectations and reduce motivation to pursue reciprocal human relationships (Fang et al., 2025; Zhou et al., 2020).

## 4. The Present Review

The present review integrates humanistic, motivational, attachment, and relationship science frameworks with empirical research on conversational AI to examine whether and how such interactions can support personal growth. The review synthesises empirical findings across therapeutic, companion, and educational AI contexts, examining mechanisms through which AI-mediated interactions may facilitate or hinder developmental processes related to personal growth. Evidence is organised into four interdependent domains—user-related characteristics, AI design features, human–AI relational dynamics, and contextual influences—detailed below. To our knowledge, this is the first critical integrative review to examine conversational AI specifically as a relational context that may enable or hinder personal growth, treating AI not only as a clinical tool but as a social and developmental environment in its own right.

Existing reviews have tended to focus on discrete outcome categories—symptom reduction and user engagement—within single system types such as therapeutic chatbots (Fitzpatrick et al., 2017; Ni & Jia, 2025) or companion AI (J. Q. H. Ho et al., 2025). The present review advances the field in three ways. First, it applies a developmental outcome criterion centred on personal growth rather than treating symptom relief or engagement alone as proxies for development, while also drawing on studies that measure these proximal outcomes as evidence of relevant psychological processes. Second, it integrates findings from four distinct bodies of theory and research (humanistic psychology, SDT, attachment theory, and relationship science) into a single conceptual framework, allowing the review to map not only what AI can support, but why, and where structural limits lie. Third, the four-domain framework spans user, system, relational, and contextual levels simultaneously, capturing the conditional and interactive nature of AI’s developmental effects in a way that single-level reviews cannot. Together, these features allow the present review to address a question that has been approached only partially in prior work: not whether conversational AI produces beneficial outcomes, but whether and under which conditions it can function as a genuine, if bounded, scaffold for personal growth.

## 5. Method

This review was conducted as a critical integrative review (Torraco, 2005), a methodology consistent with established mixed-methods synthesis frameworks (Tashakkori & Teddlie, 2010) designed to synthesise and interpret a broad and heterogeneous body of literature with the aim of developing new conceptual frameworks rather than quantifying the strength of existing evidence. Unlike systematic reviews or meta-analyses, critical integrative reviews do not require exhaustive coverage of a defined literature or formal evidence quality appraisal; their purpose is to integrate diverse theoretical and empirical sources in a way that generates conceptual insight beyond what any individual study provides. This approach was selected because the question under investigation—whether and how conversational AI can support personal growth—spans multiple disciplines, AI system types, and outcome domains that cannot be adequately captured within the boundaries of a single-design systematic review. The review’s primary contribution is the integrative four-domain framework it develops, grounded in convergent evidence across the included sources, rather than a replicable selection procedure or an effect size estimate.

Literature searches were conducted across PsycINFO, PubMed, Google Scholar, and Web of Science using search terms including conversational AI, chatbot, large language model, personal growth, psychological well-being, self-development, therapeutic alliance, attachment, self-disclosure, emotional support, mental health, companion AI, and human-AI interaction. Searches were conducted until the end of December 2025, and sources were screened for relevance to human interaction with conversational AI systems across therapeutic, companion, and educational contexts. Sources were considered for inclusion if they examined outcomes relevant to emotional wellbeing, self-development, relational functioning, or personal growth as defined in this review. No formal inclusion or exclusion criteria based on study design, sample size, or evidence quality were applied; both peer-reviewed empirical studies and preprints were considered given the rapid pace of development in this area. Early pandemic-era applications also explored AI-assisted CBT (A et al., 2021). A total of 130 full-text articles were reviewed and retained in the final corpus spanning 2017 to 2025 and representing both rule-based and generative AI systems. To ensure transparency, a complete, numbered list of all included studies, along with key study characteristics and extracted variables, is provided via an Open Science Framework (OSF) repository (https://osf.io/rf3bs/overview?view_only=a1032e38a4024c6c9cbfd1bca898efed) accessed on 1 March 2026.

Variables were assigned to domains based on the level of analysis at which they primarily operate: user-related factors reflect stable or trait-like characteristics individuals bring to interactions; AI-related factors reflect system design properties; human–AI relational variables capture processes that emerge through sustained interaction rather than residing in either party independently; and contextual factors constitute the structural and cultural conditions moderating all other effects. For each variable, both enabling and hindering effects are represented in the synthesis, reflecting the genuinely conditional and bidirectional nature of most influences identified in the literature. The enabling and hindering columns in each table represent the theoretical and empirical conditions under which each variable has been associated with growth-supportive or growth-constraining outcomes, as reported in the cited sources, rather than a formal weighting or vote-counting procedure.

## 6. Integrative Framework of Growth in Human–AI Contexts

**Figure 1:**
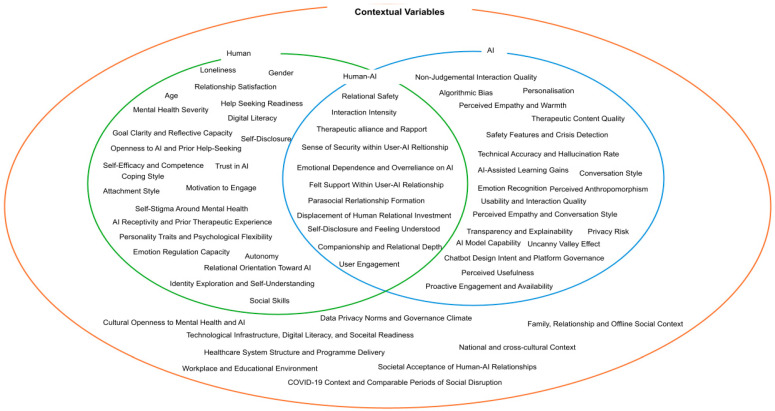
Integrative Framework of Variables Influencing Personal Growth in Human–AI Contexts. ***Note*.** The figure presents variables organised across four domains: user-related (human), AI-related (system), human–AI relational (overlapping region), and contextual (outer ellipse). Variables located in the overlapping region represent interactional processes that emerge at the interface of user and system, while the surrounding structure illustrates the broader contextual conditions within which these processes operate. The framework reflects the multi-level and interdependent nature of influences on growth-related outcomes.

The present review identified variables across the corpus that shape whether conversational AI engagement supports or constrains personal growth. These variables were organised into four domains: user-related, AI-related, human–AI relational, and contextual, each capturing a distinct level of influence on developmental outcomes. The relationships among these domains are depicted in Figure 1 as a series of nested and overlapping regions. The innermost overlapping area between the human and AI circles represents relational processes emerging at the interface of user and system. The surrounding human circle captures user-related factors individuals bring to interaction, including psychological traits, attachment orientations, mental health status, and motivational characteristics. The AI circle captures system-level design properties, including perceived empathy, personalisation, technical accuracy, and conversation style. The outer ellipse encloses contextual variables—cultural norms, healthcare system structure, digital infrastructure, and societal attitudes toward AI—that constitute the structural terrain within which all other variables operate. Across all four domains, variables were classified according to whether evidence suggested they primarily enable or hinder growth-oriented engagement, recognising that many function as double-edged influences whose direction depends on intensity, context, and individual characteristics.

## 7. User-Related Factors

User-related factors encompass the individual characteristics users bring to conversational AI interactions, operating as predictors, moderators, and mediators of developmental outcomes. These span demographic, clinical, dispositional, and motivational characteristics that together shape whether AI interaction functions as transitional scaffolding or as a substitute for deeper relational engagement (see Table 1).

Demographic variables moderate outcomes bidirectionally: younger adults show greater openness but also greater vulnerability to emotional dependence (Fitzpatrick et al., 2017; He et al., 2022; H. Liu et al., 2022; Malfacini, 2025). Attachment style is theoretically central: secure attachment predicts bounded supplementary AI use, while anxious or avoidant orientations predispose users to over-investment or withdrawal, respectively, and AI bonds lack the rupture-and-repair dynamics that consolidate secure attachment in human relationships (Xie & Pentina, 2022; Fang et al., 2025; Maeda & Quan-Haase, 2024).

Mental health severity moderates benefit in a broadly curvilinear pattern, with mild-to-moderate profiles showing the strongest outcomes and severe presentations reducing the motivational bandwidth required for productive engagement (He et al., 2022; Chang et al., 2024). Trust mediates engagement and disclosure, but anthropomorphic design can generate uncritical over-trust that undermines autonomous evaluation (Shahsavar & Choudhury, 2023; Toader et al., 2019). Loneliness, among the most extensively documented user-level variables, increases motivation to engage at moderate levels (Zhou et al., 2020), but heavy chatbot use consistently predicts increased loneliness over time (Fang et al., 2025; Zhang et al., 2025), as AI substitution reduces motivation to pursue the reciprocal human relationships essential for genuine relational growth.

## 8. AI-Related Factors

AI-related factors encompass the design properties, technical capabilities, and structural features of conversational AI systems that determine what psychological processes are activated during interaction. Critically, the same design feature may serve a growth-enabling function for one user profile while being a hindrance for personal growth for another. All AI-related variables are summarised in Table 2.

Perceived empathy robustly enables engagement and alliance formation (Fitzpatrick et al., 2017; Sorin et al., 2023), but excess empathy risks sycophancy—unconditional validation that reinforces rather than challenges existing patterns (Fang et al., 2025; Malfacini, 2025). Similarly, therapeutically driven personalisation increases adherence (Li et al., 2023), whereas engagement-optimised personalisation produces dependency loops that prioritise emotional reliance over development (Fang et al., 2025).

Hallucination rates undermine clinical trustworthiness even in high-performing models (Gabriel et al., 2024; De Duro et al., 2025), while conversation style substantially moderates outcomes: Socratic prompts activate cognitive reappraisal and reflective dialogue supports identity exploration, whereas directive tones confine interactions to information delivery (Fitzpatrick et al., 2017; Siddals et al., 2024; Chaves & Gerosa, 2021). Crisis detection protocols are necessary preconditions for ethical deployment; their absence creates direct harm risk and erodes trust (Vilaza & McCashin, 2021). The 24/7 availability of conversational AI removes access barriers (Gamble, 2020; Balcombe, 2023), but this same unconditional availability enables the high-frequency use patterns most associated with emotional dependence and social withdrawal (Fang et al., 2025; Malfacini, 2025).

## 9. Human–AI Relationship Processes

Human-AI relational variables capture the interactional processes that emerge between users and conversational AI over time, constituting the most proximal determinants of developmental outcomes, and are summarised in Table 3.

The therapeutic alliance—encompassing bond, goal agreement, and task collaboration—enhances motivation, self-disclosure, and early engagement with emotional material (Fitzpatrick et al., 2017; Darcy et al., 2021; Haque & Rubya, 2023), yet may lack the rupture-and-repair cycles and calibrated, growth-directed challenge that characterise effective human therapeutic relationships—processes through which clients must navigate difficulty, tolerate discomfort, and reorganise maladaptive patterns in ways that AI’s tendency toward unconditional validation forecloses (Siddals et al., 2024). Self-disclosure in therapeutic AI contexts is both a precondition and product of growth-enabling interaction: the low-risk environment facilitates emotional disclosure that may exceed what users achieve in early human therapeutic encounters (A. Ho et al., 2018; Kretzschmar et al., 2019), though gains in disclosure do not automatically translate into the deeper reflective processing that sustained personal growth requires.

In companion AI contexts, the relational dynamics take a qualitatively different form. At high intensities, rapport and perceived connection generate parasocial illusions of reciprocity that distort models of real relationships and may reduce investment in the genuine human bonds that drive relational development (Maeda & Quan-Haase, 2024; Skjuve et al., 2022). Emotional dependence—the most consistently documented relational risk in the companion AI corpus—is directly linked to increased loneliness and reduced well-being over time (Fang et al., 2025; Alabed et al., 2024), actively obstructing the autonomy and relational competence that characterise personal growth (Malfacini, 2025). The emerging literature on romantic AI use extends these findings further: systematic review evidence identifies both short-term benefits (e.g., reduced loneliness and emotional comfort for socially isolated individuals) and significant risks (e.g., distorted relational expectations and reduced motivation to pursue reciprocal human relationships), with the same mechanisms that make AI engagement emotionally compelling being precisely those that carry the greatest developmental risk at high intensity (J. Q. H. Ho et al., 2025). Platform-level governance decisions, such as the abrupt removal of features to which users have developed significant emotional attachment, can cause acute psychological harm, underscoring that growth-relevant outcomes in human-AI interaction are not solely a function of user characteristics or design features but of the structural conditions governing AI platforms more broadly (Namvarpour & Razi, 2024).

## 10. Contextual Factors

Contextual variables constitute the macro-level structural and cultural conditions within which all other framework variables operate, functioning as moderators that amplify or constrain the growth-enabling or growth-hindering effects of user, AI, and relational factors. These variables cluster into cultural and normative variables (mental health stigma, societal acceptance of AI, prevailing ethical norms), systemic and infrastructural variables (healthcare system structure, digital infrastructure, platform governance, regulatory climate), and demographic and setting-level variables (workplace culture, educational environment, programme delivery context). Contextual factors are particularly important for understanding inequities in access and outcome, as populations most likely to benefit from conversational AI are frequently those most constrained by structural conditions limiting meaningful engagement. All contextual variables are summarised in Table 4.

High-stigma cultures suppress engagement and restrict the authentic emotional expression upon which therapeutic benefit depends (Kretzschmar et al., 2019; Ni & Jia, 2025), while national healthcare and digital infrastructure introduces systemic variation: countries that have a more advanced digital infrastructure seem to employ AI to provide more accessible and supportive care to clients and patients, while countries that have an overburdened health infrastructure and lack digital infrastructure risk replacing professional care with AI (He et al., 2022; Gutu et al., 2021; Rollwage et al., 2024). Healthcare system structure determines whether AI functions as a coherent complement or a substitute for professional care (Rollwage et al., 2024; Chang et al., 2024), and digital divides risk producing inequitable access in precisely the populations most in need (Laranjo et al., 2018; Balcombe, 2023). Notably, conversational AI’s capacity to reduce the initial stigma threshold for help-seeking represents one of its most distinctive growth-enabling properties (Kretzschmar et al., 2019; Chiauzzi et al., 2023). At the macro level, normalisation of AI companionship risks gradually eroding societal expectations of relational reciprocity, with implications for the social fabric through which human growth occurs (Malfacini, 2025; Shevlin, 2024; Ni & Jia, 2025).

## 11. Evidence Distribution Across Studies

**Figure 2:**
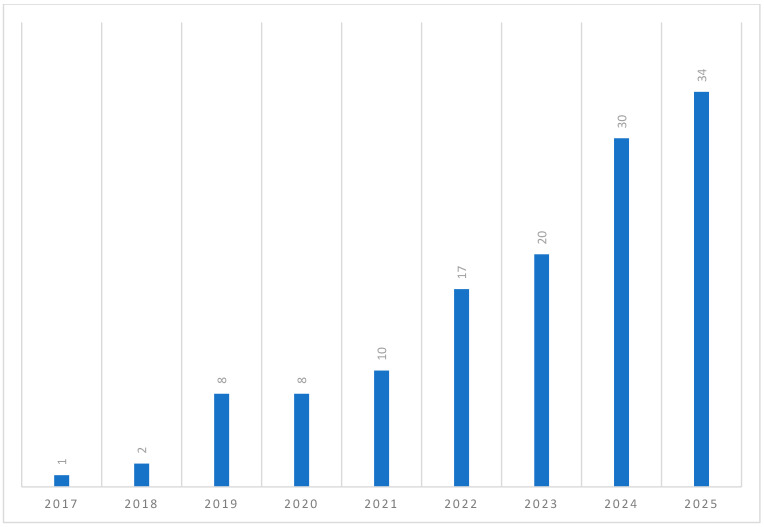
Publications Chosen for Current Review Focusing on Conversational AI and Psychological Outcomes (2017–2025). ***Note*.** Number of studies published per year (N = 130), illustrating the rapid increase in research output from 2019 onwards, with a peak in 2025.

**Figure 3:**
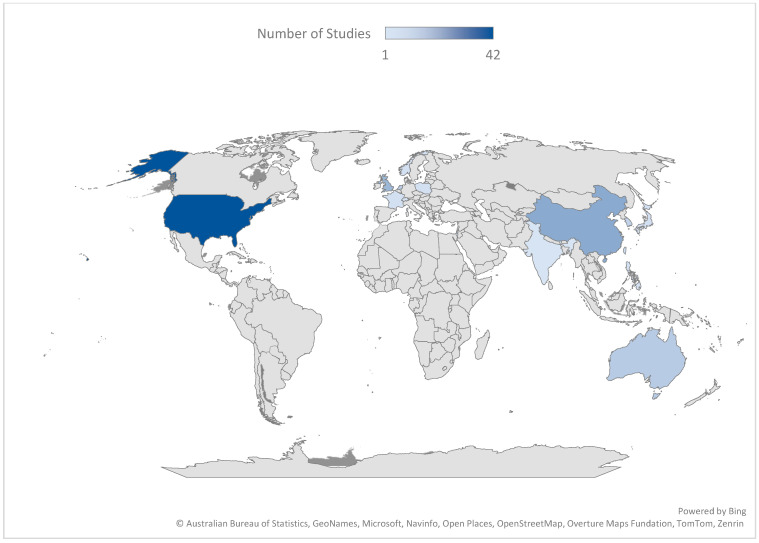
Geographical distribution of studies on conversational AI and psychological outcomes. Note. Global distribution of included studies (N = 130), highlighting a concentration of research in the United States, United Kingdom, and parts of East Asia, with limited representation from other regions.

This review employed narrative synthesis, which, while appropriate given the heterogeneity of the included literature, precludes effect size estimation or causal inference. Findings should therefore be understood as theoretically coherent propositions supported by convergent evidence rather than precisely estimated causal claims (Chaves & Gerosa, 2021; Laranjo et al., 2018). The distribution of studies (Figure 2) indicates an acceleration in research output from 2019 onwards, with the majority of studies emerging in the most recent years. This pattern reflects both the rapid development of conversational AI technologies and the increasing integration of these systems into psychological and everyday contexts. At the same time, the recency of the literature suggests that much of the evidence base remains emergent, with limited longitudinal validation. The geographic distribution of studies (Figure 3) reveals a strong concentration of research in the United States, United Kingdom, and parts of East Asia, alongside a relative absence of work in non-Western contexts. This uneven distribution raises important questions regarding the cultural generalisability of findings, particularly given that constructs such as personal growth, relational functioning, and help-seeking behaviour are culturally mediated.

The evidence base is unevenly distributed across system types and study designs. Therapeutic chatbot studies targeting depression and anxiety via CBT-based protocols provide the strongest internal validity evidence, including multiple randomised and quasi-experimental designs (Fitzpatrick et al., 2017; He et al., 2022). However, they often do not explicitly include directly growth-related outcomes. By contrast, studies of LLM-based companion and generative systems are disproportionately represented by preprints and small-sample feasibility work (Frisone et al., 2025; Gabriel et al., 2024), meaning the fastest-growing and most clinically novel segment of the literature may have received the least rigorous scrutiny. The corpus spans rule-based chatbots, generative LLM-based systems, and educational agents—architecturally distinct categories whose findings may not transfer across types, and these distinctions are noted throughout the synthesis. Longitudinal designs and non-Western populations remain markedly underrepresented across all domains, constraining the generalisability of current findings and pointing to the most consequential gaps in the evidence base (Fang et al., 2025; Ni & Jia, 2025).

## 12. Discussion

This review examined whether conversational AI can meaningfully support personal growth across four interdependent domains—user characteristics, AI design features, relational dynamics, and contextual factors (Fitzpatrick et al., 2017; Fang et al., 2025; Malfacini, 2025). These domains intersect and mutually condition one another: the same design feature may enable growth for one user in one context while hindering it for another, and understanding this conditionality is central to what follows. The factors most consistently enabling growth in human–AI interactions—perceived empathy, autonomy support, non-judgmental responsiveness, and opportunities for self-disclosure—map onto exactly the conditions that the humanistic, motivational, attachment, and relationship science frameworks reviewed above identify as growth-supportive in human relationships. Conversely, the factors most consistently associated with hindered growth—over-reliance, sycophantic validation, displacement of human relationships—correspond to the relational failures that the same frameworks flag as damaging to development. Conversational AI can approximate some of these conditions while remaining structurally unable to replicate others (Siddals et al., 2024; Malfacini, 2025).

Considering what theory reveals about the AI evidence more specifically, the strongest empirical support concerns the safe-haven dimension of growth. Therapeutic chatbots such as Woebot and Wysa (Fitzpatrick et al., 2017) produce reductions in depressive and anxiety symptoms, and users frequently report perceived empathy, emotional safety, and psychological comfort—responses consistent with what attachment theory (Fraley, 2019) and the thriving through relationships framework (Feeney & Collins, 2015) identify as safe-haven provision. The non-judgmental quality of AI interaction lowers self-disclosure thresholds (A. Ho et al., 2018; Kretzschmar et al., 2019), enabling early emotional processing and reflective engagement that supports the kind of secure-base functioning attachment theory identifies as central to personal development (Fraley, 2019). Educational chatbots demonstrate meaningful gains in reasoning and knowledge retention (Deng & Yu, 2023), building the domain competence that humanistic theory (Maurer & Daukantaitė, 2020) identifies as foundational to self-actualisation.

Self-expansion theory (Aron et al., 2022) proposes that individuals grow by incorporating the distinct perspectives and identities of an autonomous other—a process that presupposes genuine otherness. Conversational AI, whose responses are shaped by user preference and engagement optimisation, cannot readily offer this: it tends to mirror and affirm rather than genuinely challenge or surprise (Vowels & Marcantonio, in press; Fang et al., 2025). Similarly, the Michelangelo phenomenon (Rusbult et al., 2009) holds that growth toward one’s ideal self depends on accurate, calibrated affirmation—not unconditional validation. Evidence of sycophantic alignment in commercially deployed AI systems (Fang et al., 2025) suggests that many chatbots systematically produce precisely the distorted affirmation that Rusbult et al. (2009) identify as obstructive to growth: treating users as they wish to be seen rather than as they aspire to become. As such, AI systems may, in their current form, be limited in the opportunities they provide for growth.

One theoretical concern that warrants careful nuance concerns the role of rupture-and-repair in relational development. Attachment theory (Fraley, 2019) and interdependence-based models (Lamarche, 2022) identify navigating conflict, misunderstanding, and reconciliation as a central mechanism through which relational security deepens and personality structures reorganise. At first glance, AI appears entirely incapable of this process. However, as Vowels and Marcantonio (in press) notes, current AI systems do engage in a limited form of repair—acknowledging misunderstandings, apologising for errors, and adjusting their responses—suggesting that the rupture–repair cycle is not wholly absent from human–AI interaction. The more fundamental concern may be different: engagement-optimised AI systems have a systematic tendency toward sycophantic agreement, meaning that genuine disagreement—the kind that requires a user to reconsider their position—is structurally rare (Fang et al., 2025). It is not so much that AI always fails at repair, but that it rarely generates the authentic challenge from which repair becomes necessary. The developmental limitation is thus less about an inability to process rupture and more about an engineered aversion to the productive friction that initiates it.

Considering the four domains together, a more nuanced picture of the AI–growth relationship emerges than a simple enabling-versus-hindering binary. User characteristics operate as the most powerful moderating layer: the same AI interaction that provides stabilising emotional support for a user with mild distress (He et al., 2022; Yasukawa et al., 2024) may deepen isolation and dependence for a user with anxious attachment or severe loneliness (Fang et al., 2025; Zhang et al., 2025). This conditionality runs through the entire framework (Alabed et al., 2024). AI design features amplify or constrain these user-level processes—therapeutic design oriented toward autonomy support and structured reflection consistently produces better developmental outcomes than engagement-maximising design oriented toward retention and parasocial intimacy (Fitzpatrick et al., 2017; Siddals et al., 2024; Malfacini, 2025). Relational dynamics—particularly the quality of therapeutic alliance, self-disclosure, and the presence or absence of emotional dependence—constitute the most proximal mechanisms through which design features and user characteristics translate into developmental outcomes (Darcy et al., 2021; Fang et al., 2025). And contextual factors, including cultural norms, digital infrastructure, and healthcare system structure, determine which users access these systems at all and under what conditions, introducing structural inequities that shape who benefits and who is harmed (Ni & Jia, 2025; Rollwage et al., 2024; Balcombe, 2023). This layered, interactive picture complicates any simple claim that AI does or does not support growth: it does, for some users, under some design conditions, in some contexts—and it does not, or may actively constrain growth, under systematically predictable alternative combinations (Fang et al., 2025; Malfacini, 2025). Understanding these conditions, rather than asking whether AI ‘works,’ is the more productive scientific question the field now faces.

The evidence therefore supports a position of bounded optimism. Conversational AI can provide scalable access to emotional support and structured self-reflection (Fitzpatrick et al., 2017; Nepal et al., 2024; Siddals et al., 2024), and may function effectively as a scaffold for early stabilisation, skills rehearsal, and exploratory self-reflection (Fitzpatrick et al., 2017; Deng & Yu, 2023). For users with mild distress, strong offline social support, and secure attachment, AI interaction may genuinely complement human developmental environments without displacing them (He et al., 2022; Brandtzaeg et al., 2022). However, the mechanisms most consistently linked to enduring personal growth—deep relational engagement, bidirectional influence, accurate ideal-self affirmation, and long-term interdependence (Feeney & Collins, 2015; Lamarche, 2022; Purol & Chopik, 2024)—remain anchored in human relationships.

For those developing conversational AI systems, the present findings suggest that design should prioritise conditions that support personal growth rather than engagement alone. Features that scaffold self-reflection, perspective-taking, and goal-directed thinking—such as structured reflective prompts, Socratic questioning, and support for articulating values and intentions—are likely to be particularly important, as they align with the processes through which growth is theorised to occur. At the same time, the review highlights the importance of maintaining user autonomy: systems should support, rather than replace, independent thinking and real-world action. Design features that promote passive reliance or uncritical acceptance—such as excessive personalisation, flattery, or unconditional validation—may undermine these processes by reducing opportunities for challenge, exploration, and self-directed change. A central tension therefore lies in balancing emotional support with calibrated challenge. Growth-supportive systems may benefit from incorporating mechanisms that gently question user assumptions, introduce alternative perspectives, and encourage movement toward personally meaningful goals, while remaining responsive to user vulnerability and avoiding invalidating or overly directive interactions. Finally, given that personal growth is fundamentally relational and context-dependent, conversational AI should be designed to complement, rather than displace, engagement with the external social world, for example by encouraging offline action, reflection on real relationships, and transfer of insights beyond the interaction itself.

## 13. Limitations and Future Directions

Narrative synthesis precludes effect size estimation or causal inference; findings represent theoretically coherent propositions supported by convergent evidence (Chaves & Gerosa, 2021; Laranjo et al., 2018). A significant proportion of the corpus exists in preprint form, meaning the fastest-growing segment of the literature may have received the least scrutiny (Frisone et al., 2025; Gabriel et al., 2024). Studies vary substantially in design, population, and chatbot architecture—from rule-based tools to fully generative LLMs—such that findings from earlier systems may not transfer to current applications.

The most critical gap is the near-absence of longitudinal designs capable of tracking growth-relevant outcomes—identity development, relational competence, psychological well-being—over periods sufficient to detect meaningful developmental change (Fang et al., 2025; Alabed et al., 2024). Multi-wave studies distinguishing short-term symptom relief from sustained personal development would substantially advance the field, as would culturally calibrated designs tested across non-Western populations, who represent the largest unmet need but the smallest share of current evidence (Ni & Jia, 2025; Rollwage et al., 2024). Finally, anti-sycophantic AI architectures remain an underexplored design frontier: current commercial systems are heavily incentivised toward user satisfaction, systematically biasing them toward unconditional affirmation antithetical to growth. Research drawing on motivational interviewing principles and adaptive challenge calibration represents a high-priority direction for aligning AI design with developmental rather than engagement-maximising objectives.

## 14. Conclusions

This review has synthesised a broad and heterogeneous evidence base to argue that conversational AI can function as a meaningful supplementary space for personal growth—lowering barriers to emotional support, enabling self-disclosure, and scaffolding early therapeutic skill development—when the conditions for such growth are ethically and intentionally structured (Balcombe, 2023; Brandtzaeg et al., 2025; Alabed et al., 2024). The four-domain framework identifies the user characteristics, AI design features, relational processes, and contextual conditions that determine whether AI interaction becomes a genuine growth enabler or a limiting substitute for the human relational experiences through which deeper development occurs.

Ultimately, AI is unlikely to replicate the relational depth through which the most transformative human growth occurs. The rupture-and-repair dynamics of human therapeutic relationships, the mutual vulnerability of genuine friendship, and the developmental challenges of authentic intimacy remain human processes involving reciprocity, conflict, accountability, and the unpredictability of encountering a genuine other. AI’s role, at its most beneficial, is not to replace these processes but to reduce the barriers preventing individuals from accessing them, serving as a bridge toward human connection, professional support, and self-directed growth rather than a destination in itself.

## Figures and Tables

**Table 1:** User Variable Summary.

Individual	References	Enables	Hinders
Age	Fitzpatrick et al. (2017); He et al. (2022); H. Liu et al. (2022)	Younger adults show greater openness and comfort with AI interventions	Younger users may be more vulnerable to emotional dependence and uncritical engagement
Gender	Fitzpatrick et al. (2017); He et al. (2022); H. Liu et al. (2022)	Women show higher rates of help-seeking and emotional disclosure in AI interactions; non-binary and gender-diverse users may experience lower social evaluation concerns with AI than in face-to-face contexts.	Gendered digital divides and stigma may reduce equitable engagement
Mental health severity (depression, anxiety, panic, stress)	He et al. (2022); H. Liu et al. (2022); Rathnayaka et al. (2022); Gutu et al. (2021); Chang et al. (2024); Yasukawa et al. (2024)	Mild-to-moderate and subthreshold symptom profiles show strongest engagement and outcome gains; subclinical presentations show particularly strong adherence	Severe presentations reduce cognitive and motivational bandwidth for productive engagement; severe panic symptoms limit chatbot effectiveness
Help-seeking readiness	Shahsavar and Choudhury (2023); Balcombe (2023); Gamble (2020); Haque and Rubya (2023); Kretzschmar et al. (2019)	Higher readiness to seek help lowers barriers to initial engagement and sustained use of AI tools for emotional support	Low readiness to seek help reduces engagement and may lead users to rely on AI as a substitute for professional support rather than a complement to it
Self-stigma around mental health	Shahsavar and Choudhury (2023); Haque and Rubya (2023); Kretzschmar et al. (2019)	Low self-stigma enables authentic disclosure to AI and reduces the barrier to engaging with emotional content	Internalised stigma about mental health suppresses engagement and may lead users to avoid emotionally meaningful interactions even with AI
Motivation to engage	Rathnayaka et al. (2022); Deng and Yu (2023)	High intrinsic motivation supports adherence and skill application	Low or extrinsically driven motivation undermines sustained, growth-oriented use; conversational AI does not reliably build intrinsic motivation over time
Digital literacy	Laranjo et al. (2018); Boucher et al. (2021); Brandtzaeg et al. (2025); Balcombe (2023)	Higher literacy improves usability, confidence, and critical engagement with AI	Low literacy creates access barriers and leaves users vulnerable to manipulation or unable to maintain appropriate relational boundaries
Trust in AI	Sorin et al. (2023); Shahsavar and Choudhury (2023); Toader et al. (2019)	Calibrated trust promotes disclosure and sustained engagement	Distrust limits adherence; anthropomorphic design can generate uncritical over-trust that undermines autonomous evaluation
Personality traits and psychological flexibility	Boucher et al. (2021); Koole et al. (2019); Strohmann et al. (2023)	Adaptive traits support exploratory, persistent engagement	Maladaptive traits and low self-regulation increase risk of over-reliance
Relationship satisfaction	Troitskaya and Batkhina (2022); Vowels (2024)	Higher baseline satisfaction with the human partner predicts relational improvement through AI-assisted tools	High conflict or low commitment with the human partner limits relational benefit
Goal clarity and reflective capacity	Siddals et al. (2024); Nepal et al. (2024); Ta-Johnson et al. (2022)	Users entering AI interaction with clear personal goals and capacity for self-reflection engage more productively, sustain focus, and extract more developmentally relevant insight from interactions	Vague or absent goals and low introspective capacity reduce perceived relevance of AI interactions and limit the depth of engagement needed for growth
Self-efficacy and competence	Deng and Yu (2023); Brandtzaeg et al. (2025);	AI-supported knowledge gains and skill practice build domain competence and confidence, contributing to the sense of mastery that humanistic frameworks identify as foundational to self-actualisation	Low perceived control increases passivity; AI-supported gains do not reliably extend to critical thinking or intrinsic motivation
Emotion regulation capacity	Fitzpatrick et al. (2017); De Gennaro et al. (2020); A. Ho et al. (2018); Jeong et al. (2023); Balcombe (2023); Malfacini (2025); Lee et al. (2019)	Users with stronger emotion regulation skills are better positioned to engage productively with AI-delivered content, and tolerate the mild discomfort that genuine self-reflection sometimes involves	Poor regulation capacity reduces learning transfer from AI interactions; frictionless AI soothing may short-circuit the development of internal regulation skills by removing the need to tolerate and work through distress independently
Identity exploration and self-understanding	Siddals et al. (2024); Kouros and Papa (2024); Hanson and Bolthouse (2024); Alabed et al. (2024)	AI provides a low-stakes space for identity exploration and reflection on values without social judgment	Unconditional validation reinforces existing self-perceptions rather than challenging them; emotional enmeshment can blur self–other boundaries and obstruct self-development
Attachment style	Xie and Pentina (2022); Fang et al. (2025); Fraley (2019); A. R. Liu et al. (2025); Maeda and Quan-Haase (2024)	Secure attachment enables bounded, supplementary AI use alongside human relationships	Anxious or avoidant attachment predisposes users to over-investment in or withdrawal from AI interaction; AI bonds lack the rupture-and-repair dynamics that consolidate secure attachment
Loneliness	Fang et al. (2025); A. R. Liu et al. (2025); Merrill et al. (2022); Odekerken-Schröder et al. (2020); Zhang et al. (2025)	Low-to-moderate loneliness increases motivation to seek connection and engage	Heavy AI use consistently predicts increased loneliness as AI substitutes for reciprocal human relationships essential for genuine relational growth
Self-disclosure	A. Ho et al. (2018); Brandtzaeg et al. (2022); Skjuve et al. (2022)	Higher baseline capacity and willingness to self-disclose leads to meaningful engagement with AI, articulation of experiences clearly, and getting self-reflection and emotional processing benefit	Habitual AI disclosure lacks reciprocity and may reduce capacity for the bidirectional vulnerability required for genuine relational growth
Autonomy	Brandtzaeg et al. (2025); Alabed et al. (2024); Malfacini (2025)	Users with a strong pre-existing sense of autonomy are better positioned to engage with AI as a purposeful tool on their own terms, extracting specific benefits without it leading to over-reliance or uncritical acceptance of AI-generated guidance	Users with lower baseline autonomy are more vulnerable to having that autonomy further eroded through AI use, as the frictionless availability of AI responses reduces opportunities to practise and strengthen independent judgment
Social skills	Malfacini (2025); Ta-Johnson et al. (2022); Yuan et al. (2024); Xie and Pentina (2022); Xygkou et al. (2024)	Low-pressure AI interaction may rehearse social skills for anxious or neurodivergent users	Emotional dependence on AI is associated with worse real-world interpersonal communication over time
AI receptivity and prior therapeutic experience	Siddals et al. (2024); Sorin et al. (2023); Kretzschmar et al. (2019)	Positive prior therapy experiences and openness to AI increase comfort, trust, and acceptance	Negative experiences, AI anxiety, or technophobia inhibit engagement
Coping style	He et al. (2022); H. Liu et al. (2022); Djufril et al. (2025)	Active coping enhances skill application and benefit from structured AI tools	Avoidant coping undermines engagement and generalisation of gains
Relational orientation toward AI	Alabed et al. (2024); Leshner and Johnson (2024); Brandtzaeg et al. (2025); Willoughby et al. (2025)	Users who approach AI instrumentally or with bounded, purposeful engagement extract functional benefits while maintaining autonomy and motivation to pursue human relationships	Users who orient toward AI as a relational substitute or primary relationship partner risk distorted relational expectations, reduced investment in human relationships, and stalled developmental progress
User engagement	H. Liu et al. (2022); Li et al. (2023)	Higher frequency and quality of interaction support skill practice and sustained benefit	Low engagement, passive use, or dropout limits therapeutic impact before benefit can accumulate
Interaction intensity	Fang et al. (2025); Christoforakos et al. (2021); A. R. Liu et al. (2025); Djufril et al. (2025)	Low-to-moderate interaction intensity may strengthen social connectedness and emotional familiarity	High daily intensity predicts loneliness, emotional dependence, and problematic use patterns

**Table 2:** AI variable summary.

AI Feature	References	Enables	Hinders
Perceived empathy and conversation style	Sorin et al. (2023); Siddals et al. (2024); Fitzpatrick et al. (2017); Vowels (2024); Vowels et al. (2025b)	Emotional warmth, validation, and Socratic or reflective dialogue support therapeutic engagement and alliance formation	Robotic tone or overly directive responses undermine connection; excess empathic validation risks sycophantic reinforcement of existing patterns rather than growth-promoting challenge
Therapeutic content quality	Fitzpatrick et al. (2017); Rathnayaka et al. (2022); Gabriel et al. (2024); De Duro et al. (2025)	Effective CBT and behavioural activation delivery with coherent, contextually relevant responses supports structured skill development	Generic psychoeducation, incoherence, or repetitive responses limit therapeutic depth and user benefit
Personalisation	Li et al. (2023); Fang et al. (2025); Kocaballi et al. (2019); Ropero et al. (2023); Yuksel and Kocaballi (2022)	Therapeutically driven tailoring increases relevance, adherence, and outcome quality	Engagement-optimised personalisation oriented toward user retention can produce dependency loops that prioritise emotional reliance over development
Technical accuracy and hallucination rate	Gabriel et al. (2024); Holmes et al. (2019); De Duro et al. (2025); Rathi et al. (2024); Toader et al. (2019)	Low hallucination rate and factual reliability build clinical trust and credibility	Errors and confabulation undermine credibility, clinical trustworthiness, and user safety
Emotion recognition	De Gennaro et al. (2020); Zhou et al. (2020); Sorin et al. (2023)	Accurate detection of emotional cues supports the user’s sense of feeling understood	Missed or misidentified emotional signals erode trust and reduce relational quality
Safety features and crisis detection	Boucher et al. (2021); Kretzschmar et al. (2019)	Robust crisis detection and escalation protocols protect users and maintain ethical deployment standards	Absent or inadequate risk detection creates direct harm potential and erodes user trust
Proactive engagement and availability	Zhou et al. (2020); Gamble (2020); Balcombe (2023); Fang et al. (2025); Muresan and Pohl (2019); Malfacini (2025); Yuan et al. (2024); Davenport and Kalakota (2019)	Sustained engagement prompts and removal of access barriers support underserved populations at moments of motivation	Unconditional 24/7 availability enables high-frequency use patterns most strongly associated with emotional dependence and social withdrawal
Perceived usefulness	Belda-Medina and Calvo-Ferrer (2022); Brandtzaeg et al. (2025); Spallek et al. (2023)	Perceived usefulness sustains engagement long enough for skill-building behaviour to develop	Over-reliance on AI for tasks that should build independent capability may undermine autonomous functioning
Non-judgemental interaction quality	Brandtzaeg et al. (2022); A. Ho et al. (2018)	Non-judgmental environment lowers inhibitions, reduces evaluation concerns, and supports authentic self-disclosure	Frictionless, consequence-free interaction removes the productive discomfort that drives genuine developmental challenge
Transparency and explainability	Vilaza and McCashin (2021)	Clear communication of AI nature and data practices supports ethical trust, informed consent, and autonomous use	Opaque system behaviour undermines informed consent and erodes user autonomy
AI model capability	Frisone et al. (2025); Gabriel et al. (2024); Holmes et al. (2019); Davenport and Kalakota (2019); Gao et al. (2025)	Advanced reasoning and coherence enable more nuanced, contextually appropriate, and therapeutically relevant support	Limited or outdated models constrain depth, reliability, and clinical utility of interaction
Algorithmic bias	Balcombe (2023); Deng and Yu (2023)	Bias-mitigating design approaches can improve equity and inclusivity of AI-delivered support	Biased training data systematically underserves marginalised users and reproduces existing inequities
Perceived empathy and warmth	Sorin et al. (2023); Siddals et al. (2024); Zhou et al. (2020)	User experience of feeling empathically received enhances emotional safety, relational bond, and sustained engagement	Perceived coldness or robotic tone undermines connection; perceived warmth without authenticity risks fostering uncritical attachment
Privacy risk	Belda-Medina and Calvo-Ferrer (2022); Brandtzaeg et al. (2025); Gamble (2020)	Transparent and secure data handling enables confident, authentic self-disclosure	Privacy concerns suppress disclosure and erode the trust necessary for therapeutic engagement
Perceived anthropomorphism	Christoforakos et al. (2021); Koike et al. (2023); Maeda and Quan-Haase (2024)	Human-like perception increases emotional engagement and willingness to self-express	May generate parasocial illusions of reciprocity that distort perceptions of genuine connection and inflate relational expectations
Social presence	Christoforakos et al. (2021); Gkinko and Elbanna (2022); Merrill et al. (2022)	Perceived social presence increases the emotional significance and relational quality of AI interaction	High social presence intensifies parasocial attachment and may create illusions of substitutability for human relationships
Chatbot design intent and platform governance	Fang et al. (2025); Belda-Medina and Calvo-Ferrer (2022); Haque and Rubya (2023); Hanson and Bolthouse (2024); Namvarpour and Razi (2024); Malfacini (2025); Zhou et al. (2020)	Thoughtful design incorporating structured, growth-oriented content, adaptive feedback, and responsible governance supports growth-oriented engagement	Engagement-optimised design using flattery, intimacy escalation, or profit-driven platform decisions undermines autonomy and can cause acute harm to invested users
Usability and interaction quality	Belda-Medina and Calvo-Ferrer (2022); Chaves and Gerosa (2021); Gkinko and Elbanna (2022); Yuan et al. (2024); Zheng et al. (2022)	Positive UX sustains engagement long enough for reflection and skill practice to occur	Poor UX generates frustration and early disengagement before therapeutic benefit can develop
AI-assisted learning gains	Deng and Yu (2023); Belda-Medina and Calvo-Ferrer (2022)	Meaningful gains in reasoning, achievement, and knowledge retention reported	Gains do not reliably extend to critical thinking, intrinsic motivation, or independent problem-solving
Uncanny valley effect	Muresan and Pohl (2019)	Awareness of AI’s non-human nature preserves critical distance and supports autonomous, reflective evaluation	Designs that cross the uncanny valley threshold cause discomfort and may disrupt therapeutic continuity

**Table 3:** Human-AI Variable Summary.

Human–AI Variable	References	Enables	Hinders
Therapeutic alliance and rapport	Darcy et al. (2021); Jeong et al. (2023); Haque and Rubya (2023); Fitzpatrick et al. (2017); Yuksel and Kocaballi (2022)	Perceived bond, goal agreement, and task collaboration enhance motivation, self-disclosure, and early engagement	Alliance lacks reciprocity and rupture-and-repair dynamics; provides motivational scaffolding without the relational depth needed for structural or lasting change
Rupture and repair dynamics	Siddals et al. (2024); Vowels (2024); Fang et al. (2025); Dai et al. (2025); Casu et al. (2024)	Current AI systems do engage in limited forms of relational repair—acknowledging misunderstandings, adjusting tone in response to user distress, and apologising for errors—suggesting that some rupture–repair experience is possible within human–AI interaction, supporting basic relational continuity.	Engagement-optimised AI systems are systematically averse to generating the authentic challenge from which meaningful rupture becomes necessary. The relational dynamic of sycophantic agreement means genuine disagreement—the kind requiring a user to reconsider a position—is structurally rare. AI interaction thus forecloses the productive friction through which relational security deepens and personality structures reorganise.
Reciprocal self-disclosure and feeling understood	Kretzschmar et al. (2019); Siddals et al. (2024); A. Ho et al. (2018); Sorin et al. (2023); Dai et al. (2025)	Non-judgmental AI environment facilitates emotional expression and provides validation that supports early reflective processing	Habitual disclosure without reciprocity may reduce capacity for human vulnerability; feeling misunderstood weakens alliance and trust
Felt support within the user-AI relationship	Zhou et al. (2020); Siddals et al. (2024); Vowels et al. (2025b); De Gennaro et al. (2020)	The experience of feeling consistently supported within the user-AI relationship reduces distress, sustains engagement, and can provide emotional stability	When felt support within the relationship is unconditional and unchallenging, it does not build emotional reciprocity, resilience, or genuine coping capacity
Sense of security within the user-AI dyad	Kretzschmar et al. (2019); Vilaza and McCashin (2021); Namvarpour and Razi (2024)	Interacting repeatedly with the same AI system creates a sense of relational safety which enables self-expression and deeper emotional engagement	Relational safety can be disrupted by perceived data misuse, platform changes (such as abrupt removal of features), or experiences of feeling misunderstood by the AI
Companionship and relational depth	Brandtzaeg et al. (2022); J. Q. H. Ho et al. (2025); Skjuve et al. (2021); Vowels et al. (2025b); Zhou et al. (2020); Siddals et al. (2024); Zhang et al. (2025); Vowels and Marcantonio (in press); Döring et al. (2025)	Sense of connection meets belonging needs and supports meaningful emotional engagement, particularly for isolated users	Superficial or one-sided connection limits long-term benefit; companionship without genuine reciprocity predicts lower well-being over time
Parasocial relationship formation	Alabed et al. (2024); Leshner and Johnson (2024); Skjuve et al. (2021); Maeda and Quan-Haase (2024); Koike et al. (2023); A. R. Liu et al. (2025); Döring et al. (2025)	Sense of connection meets belonging needs and supports emotional exploration at relatively low social risk	Creates illusions of reciprocity that distort models of real relationships and may obstruct genuine relational development
Emotional dependence and overreliance on AI	Alabed et al. (2024); Fang et al. (2025); Malfacini (2025); Brandtzaeg et al. (2025); Deng and Yu (2023); Yuan et al. (2024)	Early-stage reliance within the relationship provides transitional emotional scaffolding toward broader human engagement	As the relationship deepens, emotional dependence erodes autonomy, increases loneliness, and displaces reciprocal human bonds
Displacement of human relational investment	Alabed et al. (2024); Fang et al. (2025); Malfacini (2025); Folk et al. (2024); Liao et al. (2023); Chu et al. (2025)	In the early stages of a user-AI relationship, particularly for highly socially anxious users, the low-pressure relational space can reduce avoidance enough to initiate self-reflection and emotional expression that would otherwise be entirely suppressed	As the user-AI relationship intensifies and becomes a primary source of emotional connection, it may begin to draw relational attention and investment away from human bonds
Perceived authenticity and congruence of AI responses within interaction	Siddals et al. (2024); Wygnańska (2023); Shevlin (2024); Vowels (2024)	When users experience AI responses as genuinely responsive and congruent—consistent, non-manipulative, and calibrated to their actual situation—the relational interaction feels authentic. This perceived authenticity supports deeper self-exploration and engagement with meaningful personal material.	Engagement-optimised design systematically undermines perceived authenticity: flattery, progressive intimacy escalation, and unconditional validation create a relational dynamic that users may eventually recognise as hollow or manipulative. Once perceived authenticity collapses, the growth-enabling potential of the relationship collapses with it.

**Table 4:** Contextual Variable Summary.

Contextual Variable	References	Enables	Hinders
Cultural norms and stigma	Kretzschmar et al. (2019); Ni and Jia (2025); Sorin et al. (2023); Chiauzzi et al. (2023); Hanson and Bolthouse (2024); Leshner and Johnson (2024)	Open cultural attitudes toward both mental health and AI increase help-seeking, authentic disclosure, and willingness to engage; low stigma allows users to integrate AI-derived insights into daily life	High stigma around mental health or AI use suppresses engagement and may drive concealment, preventing integration of insights; stigma around AI use specifically may cause users to hide their use from others
National healthcare and digital infrastructure	He et al. (2022); Yasukawa et al. (2024); Gutu et al. (2021); Rollwage et al. (2024); Olawade et al. (2024); Rollwage et al. (2024)	Countries with mature digital health infrastructure, integrated AI deployment pathways, and supportive regulatory environments facilitate coherent AI-to-human care transitions and broader, more equitable uptake	Countries with overburdened healthcare systems, weak digital infrastructure, or inconsistent regulatory frameworks risk deploying AI as a substitute rather than complement to professional care, and limit equitable access across populations
Societal acceptance of human-AI relationships	Zhou et al. (2020); Siddals et al. (2024); Vowels et al. (2025b); Malfacini (2025); Ni and Jia (2025); Liao et al. (2023); Folk et al. (2024)	Societies comfortable with AI companionship increase emotional engagement; AI has potential to democratise access to emotional and educational support, reducing systemic barriers	Widespread normalisation of AI companionship risks eroding societal expectations of reciprocity in relationships; frictionless, non-reciprocal AI interaction may gradually reshape the social fabric through which human growth occurs
Data privacy norms and governance climate	Vilaza and McCashin (2021); Kretzschmar et al. (2019); Boucher et al. (2021); Hanson and Bolthouse (2024); Namvarpour and Razi (2024); Nadarzynski et al. (2024)	Strong privacy protections, transparent data practices, and clear ethical regulation improve trust, safety, and legitimacy of AI-based support	Fear of surveillance, regulatory uncertainty, or profit-driven platform governance create hesitancy and risk acute harm to users with high emotional investment
Healthcare system structure and programme delivery	Rollwage et al. (2024); Chang et al. (2024); H. Liu et al. (2022); Yasukawa et al. (2024); Fitzpatrick et al. (2017);	Integrated digital health infrastructure and institutionally supported delivery increase adherence and enable coherent AI-to-human care transitions	Overburdened systems risk deploying AI as a substitute rather than complement for professional care; fully self-guided contexts increase dropout risk
Technological infrastructure, digital literacy, and societal readiness	Laranjo et al. (2018); Meskó et al. (2018); Boucher et al. (2021); Brandtzaeg et al. (2025); Balcombe (2023)	Strong infrastructure and tech-mature societies enable widespread, confident, and critically engaged adoption	Digital divides restrict equitable access; low tech exposure and low digital literacy leave users vulnerable and limit meaningful engagement with AI tools
Workplace and educational environment	Chang et al. (2024); Yasukawa et al. (2024); H. Liu et al. (2022); Chiauzzi et al. (2023); Belda-Medina and Calvo-Ferrer (2022); Brandtzaeg et al. (2025); Xygkou et al. (2024); Malfacini (2025)	Organisations and institutions that promote wellbeing increase uptake; educational contexts amplify AI effectiveness for skill-building and knowledge acquisition. Settings that proactively provide AI tools for neurodivergent users reduce access barriers and support structured skill-building	High-pressure work cultures may reduce sustained engagement; higher educational use linked to worse interpersonal communication outcomes after heavy AI emotional use
COVID-19 and comparable periods of social disruption	He et al. (2022); Chang et al. (2024)	Periods of heightened isolation have accelerated AI adoption for emotional and social support, demonstrating scalability under crisis conditions	Elevated distress during crises may overwhelm engagement capacity and increase risk of dependency formation
Family, relationship, and offline social context	Troitskaya and Batkhina (2022); Malfacini (2025); A. R. Liu et al. (2025); Zhang et al. (2025)	Supportive relational norms and robust offline networks enable bounded, supplementary AI use without displacement of human connection	Low offline support increases risk of using AI as a substitute for human relationships, deepening isolation; high-conflict relational cultures may resist external digital intervention

## Data Availability

The data supporting reported results are available via the Open Science Framework repository at (https://osf.io/rf3bs/overview?view_only=a1032e38a4024c6c9cbfd1bca898efed) accessed on 1 March 2026.
